# Modulating the electron-transfer properties of a mixed-valence system through host–guest chemistry†
†Electronic supplementary information (ESI) available: More experimental details, crystallographic data, ORTEP, and packing diagram, NMR titrations, Job Plots, calculated host–guest distances, SWV for addition of iodide. CCDC 1004247. For ESI and crystallographic data in CIF or other electronic format see DOI: 10.1039/c4sc02799j


**DOI:** 10.1039/c4sc02799j

**Published:** 2014-11-26

**Authors:** Ahmed Zubi, Ashley Wragg, Simon Turega, Harry Adams, Paulo J. Costa, Vítor Félix, Jim A. Thomas

**Affiliations:** a Department of Chemistry , University of Sheffield , Sheffield , UK . Email: james.thomas@sheffield.ac.uk ; Fax: +44 114 222 9346 ; Tel: +44 114 222 9325; b Biomedical Research Centre , Sheffield Hallam University , Sheffield , S1 1WB , UK; c Departamento de Química , QOPNA and Secção Autónoma de Ciências da Saúde , Universidade de Aveiro , 3810-193 , Aveiro , Portugal; d Departamento de Química , CICECO and Secção Autónoma de Ciências da Saúde , Universidade de Aveiro , 3810-193 , Aveiro , Portugal . Email: vitor.felix@ua.pt ; Fax: +351 234 370 084 ; Tel: +351 234 370 729

## Abstract

Metal directed self-assembly has become a much-studied route towards complex molecular architectures. Although studies on mixed valence, MV, systems accessible through this approach are almost non-existent, the potential applications of such systems are very exciting as MV states provide the basis of a number of molecular-scale devices, including single electron wires and switches. Furthermore, while many novel hosts for guest ions and molecules have been developed through metal directed self-assembly, as these products tend to be kinetically labile, very few electrochemical studies have been reported. Herein, we report that the interplay between the binding properties and redox activity of a self-assembled trinuclear Ru^II^ macrocycle leads to an hitherto unreported phenomenon, in which access to specific MV states can be gated by host–guest chemistry. Thus, this system is the first in which MV states and the extent of electron delocalisation are switched by an ion without any change in electrochemical potential.

## Abstract 2

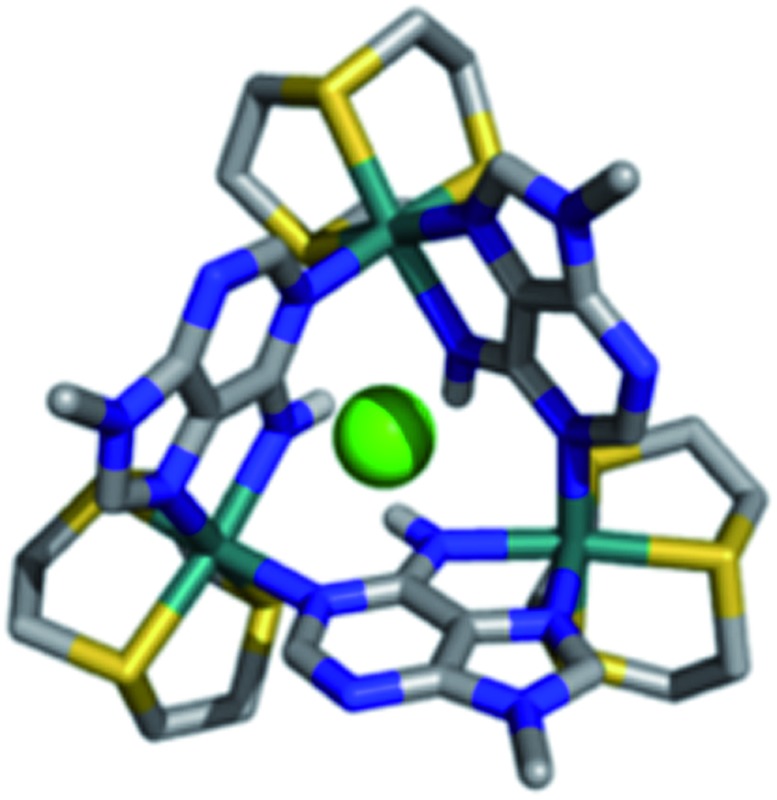
Herein, we report that the interplay between the binding properties and redox activity of a self-assembled trinuclear Ru^II^ macrocycle leads to an hitherto unreported phenomenon, in which access to specific MV states is gated by host–guest chemistry.

## Introduction

Thanks to their electrochemical and photophysical properties, oligonuclear Ru^II^-complexes have a wide variety of possible applications from abiotic light-harvesting to luminescent DNA binding substrates.^1^–^8^ The electron transfer properties of such complexes have been extensively studied; particularly Ru^III/II^ mixed valence, MV, systems. The prototype MV complex is the Creutz–Taube ion, which was first reported over forty years ago.^9^–^12^ The original reason for interest in this complex and its numerous analogues was that they provided testable experimental and theoretical models for many biological electron transfer processes. MV systems are still much studied, not least because they often function as key components in a broad range of single-molecule devices.^13^–^15^

In a handful of reports the effect of supramolecular interactions on the electronic interactions within MV states has been explored. Two studies have shown that supramolecular interactions between individual redox-active units and specific crown ethers can modulate electronic delocalisation between centres,^16^,^17^ whilst the Das group has shown that encapsulation of a ligand bridge within a cyclodextrin can enhance electron transfer rates.^18^

In separate research, metal-ion directed self-assembly has emerged as a versatile route to supramolecular architectures.^19^–^23^ Much of this work has been aimed at new hosts for ionic and molecular guests. Although the metal ion is often just a structural motif in the final assembly, its inclusion can enhance the physical properties and functionality of the host, yielding assemblies that function as sensors for specific molecular guests.^24^,^25^

Strikingly, whilst a considerable number of studies have investigated electro-active self-assembled macrocycles, virtually all this work has involved the redox properties of the organic components of such systems;^26^ there are very few reports focusing on the electrochemistry of metal ion components.^27^ Furthermore, despite the huge activity in this area, reports on metallomacrocycles containing ruthenium moieties are relatively rare, whilst only a handful of MV systems have been reported – reflecting the kinetically inert nature of such centres.^28^–^32^

**Fig. 1:**
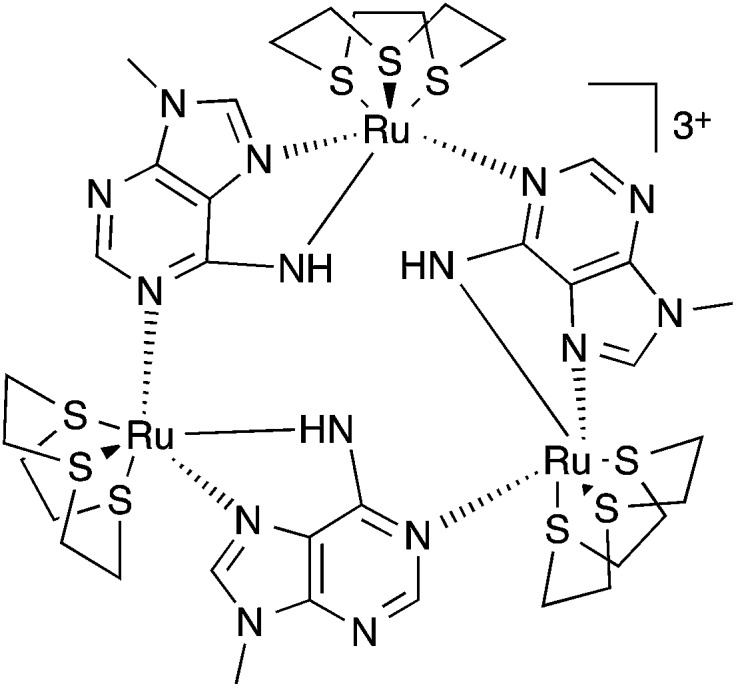
Structure of macrocycle **1**^3+^.

To combine the electron transfer properties of oligonuclear ruthenium-based MV systems with the synthetic versatility of self-assembly, we have been investigating metallomacrocycles containing embed ruthenium units. Our approach has been to either exploit the “complex-as-ligand” concept^33^–^35^ or to “labilize” inert Ru^II^ centers. Using this latter method, we have used the Ru^II^([9]aneS_3_) centre as – for a combination of steric and electronic reasons – this moiety is labile at high temperatures, but is kinetically inert at room temperature.^36^ We have demonstrated that this building block reacts with 9-methyladenine, 9MA, and other suitably hindered adenine derivatives to form metallomacrocycles such as **1**^3+^, Fig. 1, which can be reversibly oxidized into three other oxidation states, two of which are mixed valence.^37^,^38^

Most interestingly, due to the unusual connectivity of this macrocycle, it displays unique electronic properties: optical studies revealed that whilst the Ru^II^_2_Ru^III^ MV state is an electron-hopping, valence-localized, Robin and Day^39^ Class II system, the Ru^II^Ru^III^_3_ state is valence-delocalized, Class III.

Studies by the Severin group on kinetically labile [Ru(arene)]-based neutral macrocycles have resulted in analogues of 12-crown-3, that bind alkali metal ions in non-aqueous solvents with micromolar affinities.^40^,^41^ Moreover, Bedford and Tucker^42^ have shown that when the [9]aneS_3_ ligand is coordinated to a cationic metal center it can recognize anions through C–H···X hydrogen bonding interactions. Given that **1**^3+^ is cationic and possesses an array of thiacrown-based hydrogen-bonding donor groups, we reasoned that it would be a receptor for anionic guests. Herein, we describe how recognition processes involving this metallomacrocycle modulate its electronic properties in a unique manner. In particular, we report on the first MV system to display electron transfer properties that are modulated by host–guest chemistry.

## Results and discussion

### Structural studies

**Fig. 2:**
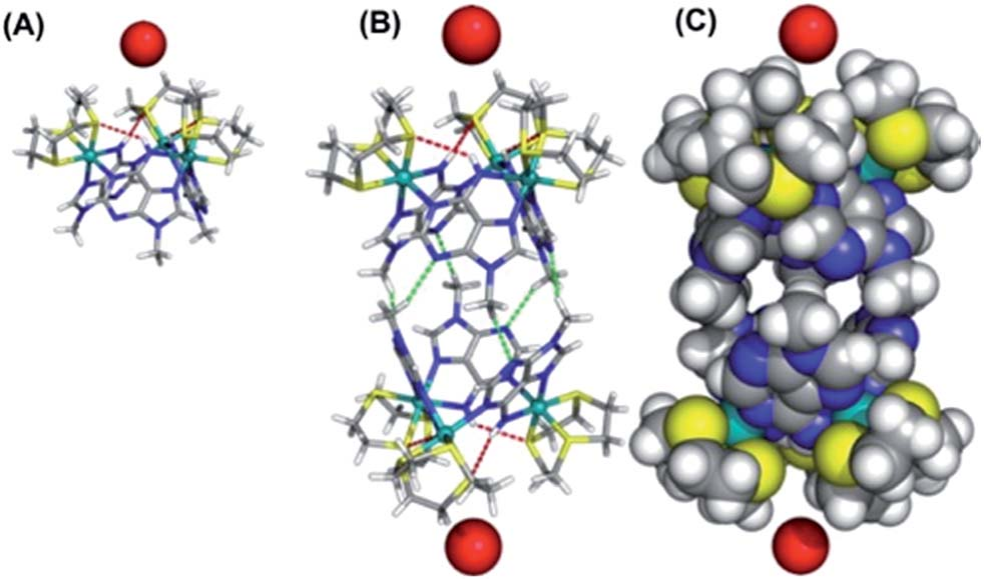
(A) Details from the single crystal X-ray structure of [**1**](Br)_3_ showing the weak N–H···S hydrogen bonds (red dashed lines) within the of **1**^3+^ unit. (B) Dimeric “capsules” formed in the structure through C–H···N hydrogen bonds (green dashed lines) between facing bowls. (C) CPK model of capsules showing axial entrances occupied by bromide anions and equatorial “gates” locked by the steric bulk of *N*-methyl groups of 9MA ligands. The following atomic colour scheme was used: carbons in grey, hydrogens in white, sulfurs in yellow, nitrogens in blue, ruthenium centres in teal and bromide anions in red.

We attempted to crystalize the macrocycle with a variety of anions in a number of different solvent systems, finally obtaining X-ray quality crystals of [**1**](Br)_3_ – Fig. 2. The structure's asymmetric unit is composed of two **1**^3+^ cations (A and B) and six bromide counter-ions (for the ORTEP diagram, see the ESI†). In cation A two [9]aneS_3_ ligands are disordered over alternative positions, while B only has one disordered thiacrown. Aside from these structural features, A and B are equivalent as illustrated by the bond lengths and angles (Table S2 in the ESI†). The metallomacrocycle has two possible binding pockets. An α pocket defined by the thiacrown ligands and the N–H binding sites from 9MA units, and a β pocket, defined by 9MA bridging ligands projecting out to give a bowl shape aromatic surface – Fig. 2A.

Pairs of bowls, related by a crystallographic inversion centre, create capsule-like structures in which two facing β pockets define the capsule cavity. This structure is formed by an array of weak C–H···N hydrogen bond – see Fig. 2B. In the B-based capsules, there are three independent H···N interactions. While for the A-based capsules, two H···N interactions are observed, the third H···N distance of 2.82 Å being longer than the sum of atomic van der Waals radii. A space-filling representation of the capsule, Fig. 2C, shows that its equator is defined by *N*-methyl groups. Notably, the two α pockets of individual bowls are occupied by bromide counter-ions. These two anions are held at a distance of 5.20, 5.43 and 5.62 Å respectively from the three N–H binding sites of A-based capsule and 5.24, 5.32 and 5.64 Å from the analogous residues of the B-based capsule. At higher levels, the capsules form alternating linear strands (see Fig. S2 in the ESI†). Given evidence of host–guest interactions in the solid state, the interaction of **1**^3+^ with anionic guests in MeCN solution was investigated.

### NMR spectroscopic studies

**Fig. 3:**
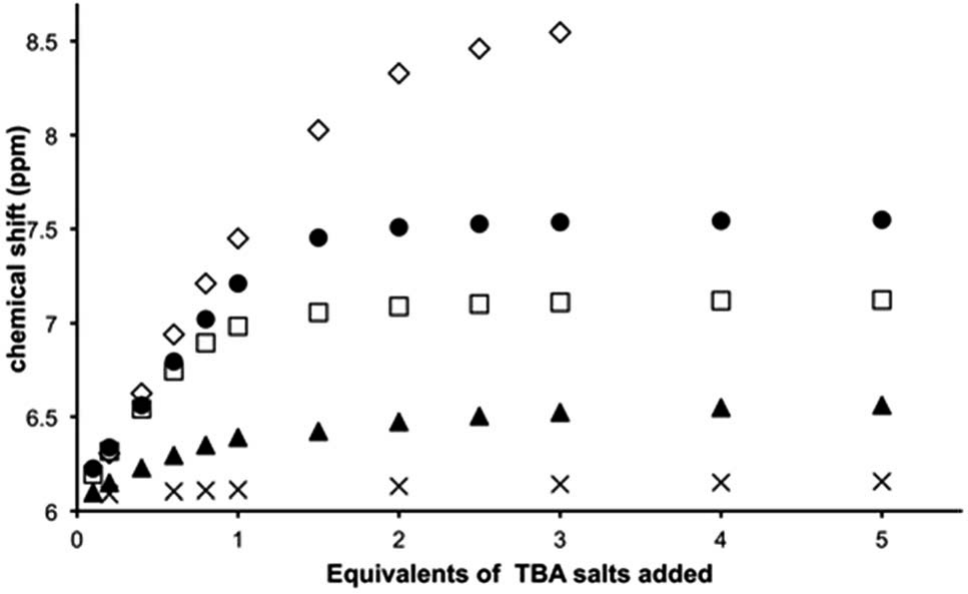
Changes in the chemical shifts of the 9MA-based NH6 proton of **1**^3+^ (concentration of hexafluorophasphate salt: 1.75 mM l^–1^ of host dissolved in d^3^-MeCN) on addition of the following selected anions: ClO_4_^–^ (×), I^–^ (▲), Br^–^ (□), Cl^–^ (), and F^–^ (). The data for the fluoride titration is truncated as higher binding ratios lead to precipitation of the host.

Titrations reveal that addition of specific anions produce changes in the 9MA-based protons signals of [**1**](PF_6_)_3_. Whilst no shifts are seen for 9-methyl hydrogens, amino group protons (NH6) display downfield shifting as depicted in Fig. S3† for the titration with TBACl salt (see ESI†); furthermore these shifts are dependent on the guest. Whilst ClO_4_^–^ induces a maximum downfield shift of 0.115 ppm, equivalent concentrations of halide ions produce much greater effects – Fig. 3.

The largest shift – of 3.651 ppm – is observed with fluoride ion; these shifts reflect the high polarizing effect of fluoride anion, indeed at higher mixing ratios still – at which precipitation begins to occur and the NH6 proton signal considerably broadens – it is clear that the host is at least partly deprotonated. Job plots using the NMR changes revealed that receptor **1**^3+^ binds to all the anionic guests investigated in a 1 : 1 ratio – Fig. S4 in the ESI.† Given this stoichiometry, and the pattern of shifting for the 9MA-based protons, we conclude that in solution anion guests are bound within the β pocket of the host. Support for this conclusion is also provided by large shifts in thiacrown-based protons.

The changes for [9]aneS_3_ protons are more complex with up and downfield shifts being observed. The crystal structure data shows close contacts between some ethylene protons of the thiacrown ligands and anionic guests; the strength of this interaction is reflected in the downfield shifting of these protons. However, a second effect also affects the thiacrown-based signals. Due to sterics, binding to this site will reduce the conformational flexibility of the coordinated thiacrowns and concomitantly increase the rigidity of the receptor; this effect will be greatest for protons on the interior of the binding pocket. A close inspection confirms this hypothesis revealing that – due to decreased fluxionality – several multiplets split into simpler signals as shown in Fig. S5† for chloride. Again, these effects are dependent on the nature of the guest: for perchlorate the biggest shift in thiacrown signals is around 0.15 ppm, while changes of almost 0.5 ppm are observed in titrations with iodide.

Using NH resonances shifts to fit to a standard 1 : 1 binding model, association constants were calculated – Table 1. The data reveal that **1**^3+^ binds to Cl^–^ and Br^–^ up to almost three orders of magnitude more strongly than other ions and that Cl^–^ is bound with the highest affinity (*K*_a_ > 10^5^ M^–1^). The values for Cl^–^ and Br^–^ are likely to be lower limits as – due to the concentration regime employed – NMR titrations only provide accurate estimates of *K*_a_ for weak or intermediate interactions. Absorption spectroscopy-based titrations were not possible as no guest-induced little change in macrocycle spectrum was observed.

**Table 1:** Estimates of binding affinities for selected halide ions derived from the observed NMR shifts

Guest anion	*K*_a_ [M^–1^]
F^–^	2.83 × 10^2^
Cl^–^	1.56 × 10^5^
Br^–^	3.92 × 10^4^
I^–^	2.09 × 10^3^

### Theoretical studies

**Fig. 4:**
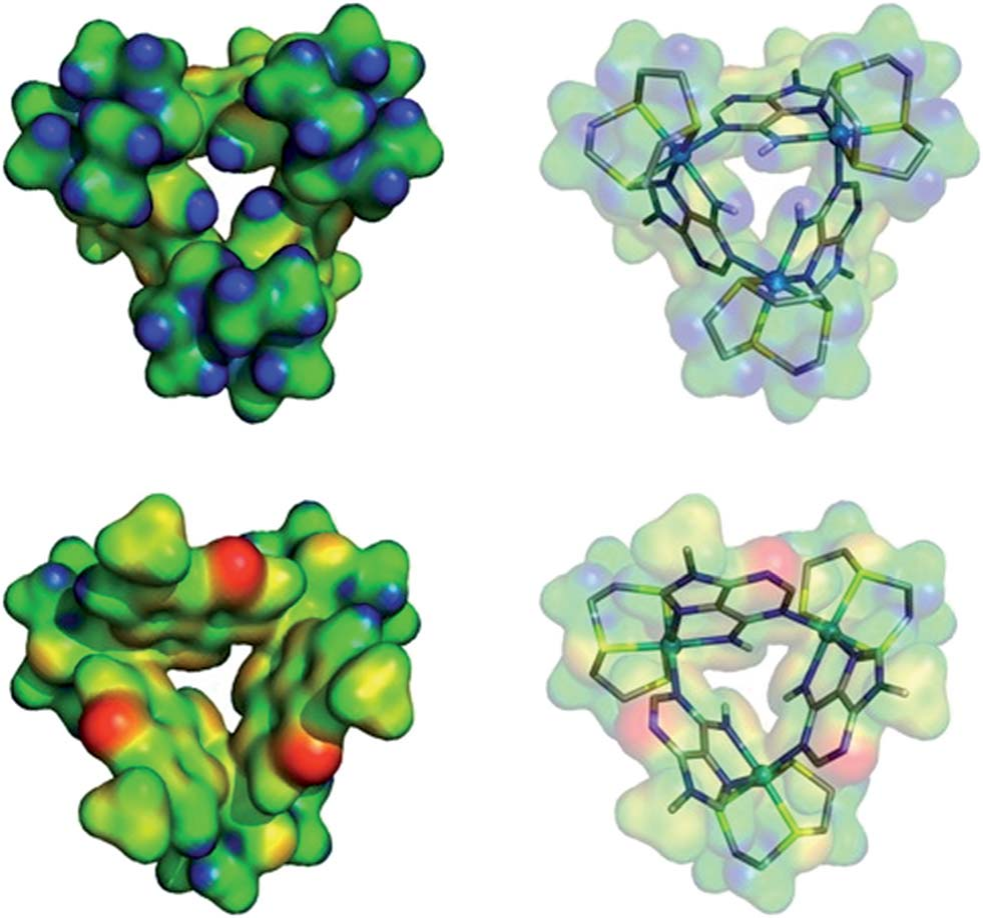
Electrostatic potential mapped on the molecular electron density surface (0.02 electrons per Bohr^3^) for receptor **1**^3+^ presented in two different views. (Top) N–H binding site pointing to front. (Bottom) 9-Methyl adenine groups pointing to front. The color scale runs from 0.10 (red) to 0.48 (blue) atomic units.

The computed electrostatic potential of the anion-free host mapped onto the molecular electron density surface, Fig. 4, reveals the α site clearly has the most positive electrostatic potential further indicating the α pocket is the preferred anion binding site. Therefore all the experimental and theoretical data indicate that anion binding at the β site can be discounted.

**Fig. 5:**
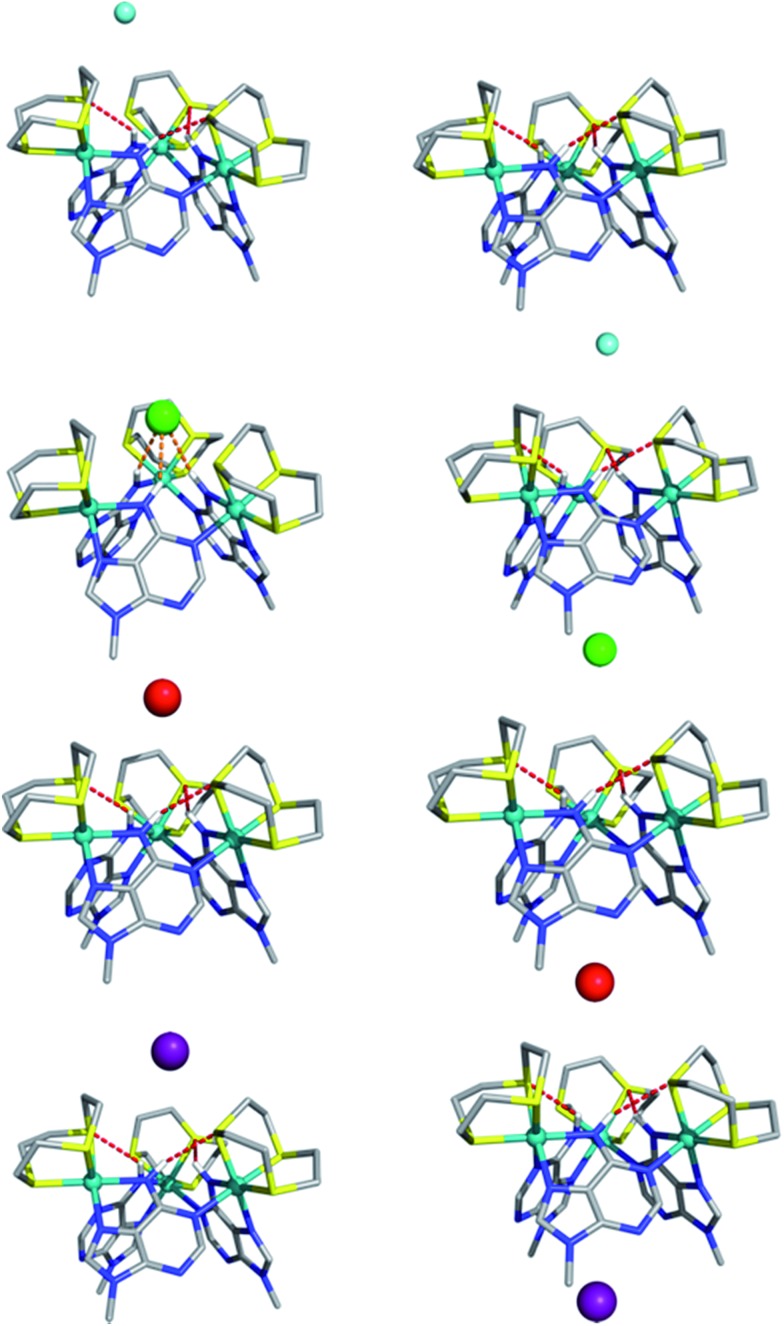
Optimized structures of **1**^3+^ with F^–^ (cyan), Cl^–^ (green), Br^–^ (red), and I^–^ (purple) for binding in the α pocket (left) or the β pocket (right). Binding scenario β is disfavoured relatively to α by 3.8 (F^–^), 1.9 (Cl^–^), 2.5 (Br^–^) and 2.2 (I^–^) kcal mol^–1^ respectively. The N–H···S and N–H···Cl^–^ hydrogen bonds are drawn as red and orange dashed lines, respectively.

Calculated structures for individual halide binding in the α binding pocket are depicted in Fig. 5 and summarized in Table 2. The distances from the centre of mass, defined by the three nitrogen atoms of the N–H binding groups (COM^N^) to fluoride is quite large (5.949 Å) indicating absence of any N–H···F^–^ interactions. Only intramolecular N–H···S hydrogen bonds are present in the optimised structure. Likewise for I^–^, no intermolecular N–H···I^–^ hydrogen bonds are observed; since the H···I^–^ average distances (4.80 Å) and corresponding N–H···I^–^ angles (average = 141°) are too small compared to typical H···I^–^ values. Indeed, the coordination geometry observed in the crystal structure of [**1**](Br)_3_ indicates that the N–H moieties of all three 9MA bridging ligands cannot point to the anion simultaneously; hence hydrogen bond angles cannot optimise, leading to weaker receptor–anion hydrogen bonding interactions. Over these effects lead to the iodine being displaced from the binding pocket by a COM^N^··· distance of 5.318 Å.

**Table 2:** Relevant intermolecular distances (Å) and N–H···X angles (°, *italics*) between **1**^3+^ and the anions X = F^–^, Cl^–^, Br^–^, and I^–^ obtained from the DFT calculations

X	F^–^	Cl^–^	Br^–^	I^–^
COM^N^···X^*a*^	5.949	3.108	5.195	5.318
N–H···X^*b*^	5.15, *148*	2.70, *147*	4.69, *141*	4.81, *141*
	5.28, *119*	2.70, *146*	4.70, *140*	4.80, *141*
	5.90, *146*	2.73, *147*	4.66, *141*	4.78, *141*
N–H···S^*b*^	2.65, *130*	2.72, *124*	2.63, *130*	2.64, *130*
	2.66, *130*	2.74, *123*	2.64, *129*	2.63, *129*
	2.64, *130*	2.73, *124*	2.63, *131*	2.65, *129*

^*a*^COM^N^ refers to the centre of mass, defined by the nitrogen atoms of the N–H binding groups.

^*b*^The values given correspond to H···X or H···S distances.

In agreement with the experimental data showing a slightly higher binding affinity for iodide compared with fluoride, this distance is shorter than the equivalent COM^N^···F^–^ distance. Besides the fact that iodide is intrinsically a weaker hydrogen bond acceptor, it seems the size of the anion is too large for the metallomacrocyclic cavity, preventing stronger N–H···I^–^ hydrogen bonds. In contrast to the iodide, the fluoride anion is too small to complement the binding pocket of the macrocycle and thus a lower binding affinity for this anion is also observed.

The computational studies for chloride binding to the α pocket of the host indicate that N–H···Cl^–^ hydrogen bonds are formed with an H···Cl^–^ average distance and N–H···Cl^–^ angle of 2.71 Å and 147°, respectively. This corresponds to a COM^N^···Cl^–^ value of 3.108 Å and results in a concomitant weakening of the intramolecular N–H···S bonds. The calculated H···Cl^–^ distances are typical of N–H···Cl^–^ hydrogen bonds, although the N–H···Cl^–^ angles are substantially lower than the ideal (180°). Once again, this is due to the structure of the host preventing the three bridging ligand N–H groups from simultaneously pointing at the anion. However, clearly the size of chloride anion complements the host cavity size, leading to shorter distances and stronger host–guest interactions than those reported for other halide anions.

As mentioned above, the optimized geometry of **1**^3+^ with Br^–^ is in agreement with the X-ray crystal structure, although the calculated COM^N^···Br^–^ distance, 5.195 Å, is shorter than the experimental value (5.911 Å). However, in the crystal packing the bromide anion is “shared” by two **1**^3+^ adjacent capsule units (see ESI†), which is not the case in the calculated solution structure. Furthermore, in accordance with recognition based on the host–guest size, fitting for the H···Br^–^ distances are intermediate between the H···Cl^–^ and H···I^–^ distances.

### Electrochemical studies

As outlined above, previous studies have revealed that electronic interaction in the two MV states of the macrocycle is not the same: the [Ru^II^_2_Ru^III^] valence state (**1**^4+^) is electron hopping, whilst the [Ru^II^Ru^III^_2_] state (**1**^5+^) electronically delocalized.^37^ This is due to the distinctive molecular architecture of the macrocycle: as metal centres are connected through peripherally arranged bridging ligands, changes in the bonds and angles at one metal centre are mechanically coupled to the other two. Since binding to anionic guests often leads to the anodic shifting in the oxidation of electroactive hosts and structural changes within the such receptors,^43^–^45^ the effect of anion binding on the electrochemical properties of **1**^3+^ was then investigated.

**Fig. 6:**
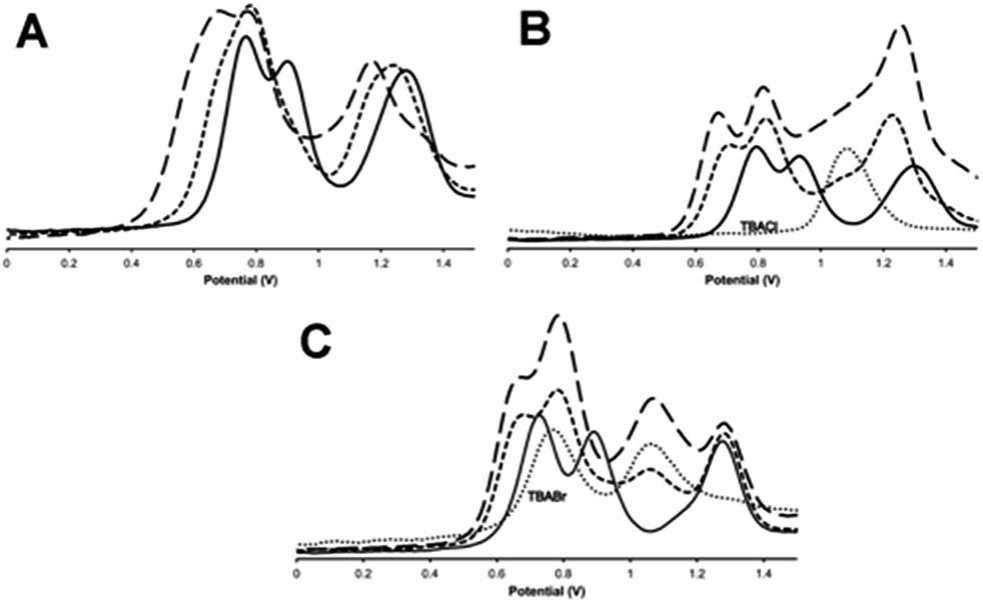
Square wave voltammograms, SWVs, for the oxidations of **1**^3+^ on the addition of (A) TBAF; (B) TBACl; (C) TBABr. Key: (—) = untreated **1**^3+^, ( <svg xmlns="http://www.w3.org/2000/svg" version="1.0" width="16.000000pt" height="16.000000pt" viewBox="0 0 16.000000 16.000000" preserveAspectRatio="xMidYMid meet"><metadata> Created by potrace 1.16, written by Peter Selinger 2001-2019 </metadata><g transform="translate(1.000000,15.000000) scale(0.005147,-0.005147)" fill="currentColor" stroke="none"><path d="M0 1440 l0 -80 360 0 360 0 0 80 0 80 -360 0 -360 0 0 -80z M1040 1440 l0 -80 360 0 360 0 0 80 0 80 -360 0 -360 0 0 -80z M2080 1440 l0 -80 320 0 320 0 0 80 0 80 -320 0 -320 0 0 -80z"/></g></svg> ) = +1 equivalent of guest, (–––) = +3 equivalents of guest. In (B) and (C) (···) = SWV of TBACl and TBABr respectively in identical conditions (*vs.* Ag/AgCl solvent: 0.1 M TBAPF_6_ in acetonitrile).

The general changes induced by anion addition on the electrochemistry of **1**^3+^ are most clearly observed using square wave voltammetry and fluoride as a non-redox-active guest – Fig 6A. As expected, on addition of fluoride, all three Ru^III/II^-based oxidation potentials are shifted anodically. Interestingly, the response of individual couples is not identical. Up to one equivalent of fluoride causes the first two oxidations to shift into each other, however they separate on further additions, resulting in a maximum Δ*E*_p_ of 100–120 mV, when three equivalents of anion are added, Fig 6A, Table 3. Further addition of anion produced no additional shifts in oxidation potential until precipitation of the host occurs. Despite analyses for other halide being complicated by the guests' intrinsic redox activity, very different effects were still delineated.

**Table 3:** Maximum Guest induced electrochemical shifts for **1**^3+^

Halide	Δ*E*_1/2_(1)/mV	Δ*E*_1/2_(1)/mV	Δ*E*_1/2_(1)/mV
F^–^	100	120	110
Cl^–^	120	115	45
Br^–^	65	∼100^*a*^	–10
I^–^	∼0^*a*^	5	0

^*a*^It is not possible to accurately estimate this value due to a close or overlapping guest-based oxidation couple.

With chloride anions, shifts in host oxidation potentials are virtually over at a 1 : 1 host : guest binding ratio – Fig 6B, additions of up to a further three equivalents of chloride ion only induce very small additional shifts. This is consistent with the high chloride binding affinity of the macrocycle. A “shoulder” between the second and third oxidation of the host also grows in as chloride is added, this is assigned to the oxidation of chloride, which occurs at 1.08 V in these conditions.^46^ It appears that this couple is broader and slightly anodically shifted compared its free value – this perturbation is likely due to the oxidation of chloride bound to the anionic host as this would be expected to shift in this way. More notably, although the first two Ru^II^ oxidations of the host are anodically shifted by similar amounts – around 120 mV – the shift for the third oxidation is less half this magnitude.

Any analysis of host-based potential shifts induced by bromide is greatly complicated by the fact that this anion is oxidized in two one-electron steps at 0.765 V and 1.065 V respectively.^47^ So whilst the first host oxidation is clearly defined – being anodically shifted by 65 mV compared to the free host – the second oxidation is difficult to deconvolute from a bromide-based couple Fig. 6c. However, most strikingly, the third oxidation is clearly *cathodically* shifted, suggesting a complex, guest-induced, redistribution of the host's electronic structure.^48^–^50^

Iodide is also oxidized in two discrete one-electron processes.^47^ Although the second of these process has almost exactly the same potential as the first oxidation of free **1**^3+^ it is clear that, even at >5 guest equivalents, no host-based oxidation shifts are observed – see Fig. S6 in the ESI.†

Using this electrochemical data, the effects of halide guest binding on the comproportionation constants, *K*_c_, for **1**^4+^ and **1**^5+^ were estimated – Table 4 – as *K*_c_ values are a direct measure of the thermodynamic stability of individual MV states.^51^ To aid comparison, *K*_c_ values for the hexafluorophosphate salt of the macrocycle in the same conditions are also included. Although the redox activity of bromide makes it difficult to make conclusion on this guest, chloride and fluoride clearly destabilize the **1**^4+^ state. Contrastingly, whilst fluoride and iodide have much less effect on **1**^5+^, chloride induces a large stability increases. Furthermore, since potential shifts for the third oxidation of the macrocycle are markers for the stability of **1**^6+^, it is clear that this redox state is most stabilized by fluoride guests, whilst the cathodic shift in the third couple induced by bromide suggests that **1**^6+^ is destabilized by any interaction with this anion.

**Table 4:** 2-Comproportionation constants for **1**^4+^ and **1**^5+^ in the presence of anion guests

Guest	*K*_c_ (**1**^4+^)	*K*_c_ (**1**^5+^)
PF_6_^–^	290	4.0 × 10^6^
F^–^	55	4.5 × 10^6^
Cl^–^	245	3.0 × 10^7^
Br^–^	—^*a*^	—^*a*^
I^–^	∼0^*a*^	1.2 × 10^6^

^*a*^It is not possible to accurately estimate this value due to overlapping host- and guest-based potentials.

The host's electrochemical response to anion binding is a product of its unique combination of properties. Since they are embedded into the macrocycle, oxidation of individual ruthenium(ii) units modulate the host's entire structure. The properties of this redox chain can be likened to those of a dynamic combinatorial library, DCL, of host architectures. In a DCL, differential host–guest interactions thermodynamically select the “best” host for a specific guest within a chemically equilibrating mixture;^52^–^54^ in the case of **1**^3+^ to **1**^6+^, the interactions select for, and stabilize, the best host redox state. Given that fluoride is a small ion with a high charge density it is not surprising that this guest stabilizes the **1**^6+^ oxidation state more than the other halide ions. However, analysis of the data also clearly indicates that chloride and bromide ions select for **1**^5+^ presumably as this is the best match of size and charge density between the host and guest. Finally the electrochemical interaction with iodide is weak as the size and charge density of this guest is mismatched to the host's well-defined binding site.

**Fig. 7:**
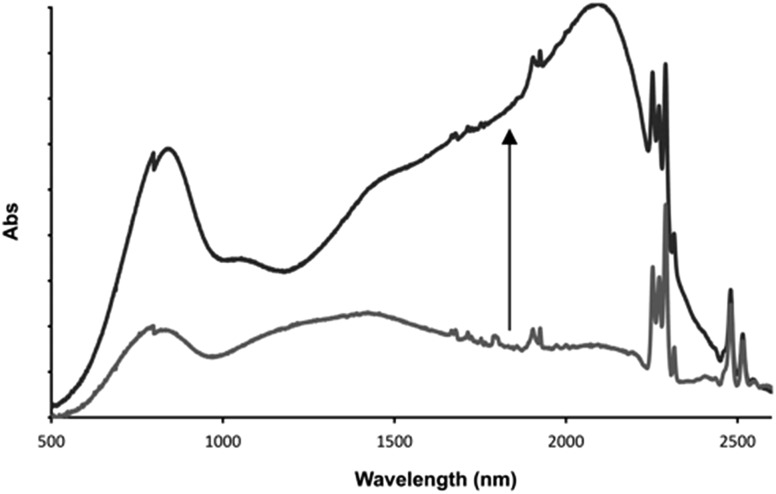
Low energy optical changes on addition of one equivalent of fluoride ion to electrochemically generated **1**^4+^ after equilibration with one equivalent of fluoride guest. Solvent: 0.1 M TBAPF_6_ in acetonitrile at 273 K.

This distinctive combination of multiple oxidation states and host–guest chemistry means that **1**^3+^ functions as a novel ion-triggered device. Through spectroelectrochemistry using a OTTLE cell, **1**^3+^ was first oxidized into its Ru^II^_2_Ru^III^ MV state (**1**^4+^) by holding it at a potential just under that required for oxidation into the Ru^II^Ru^III^_2_ MV state (0.980 V), generating the previously reported^34^,^35^ characteristic absorption spectrum with structured intervalance charge transfer (IVCT) bands in the NIR – Fig. 7.

After the addition of one equivalent of F^–^, chosen as it is not itself redox active, the solution was left for 30 minutes at the same potential. This allowed time for the anion guest to diffuse from the top of the cell to the electrode and for a new equilibrium between the electrode and oxidized product to be established. Strikingly, after this period, the IVCT bands displayed bathochromic shifting and increases in intensity; with the thiacrown(S) → Ru^III^ ligand-to-metal charge-transfer centered at ∼800 nm also growing in intensity. A comparison with previously reported data, generated in the absence of a guest but at a more positive potential, confirms that this finally generated spectrum is that of **1**^5+^. These observations confirm an anion-triggered change of MV state *without* any change in potential. Although this effect means that the host could function as a optical sensor for anions, its response can also be viewed as the operation of a Boolean logic AND gate^55^,^56^ where the two inputs are a potential difference and fluoride anion, while the output is the large NIR optical change induced by increased electronic delocalization.

## Conclusions

**1** ^3+^ displays selective binding to specific halide anions, which induce characteristic shifts in the host's Ru^II^-based oxidation potentials. This facilitates a new phenomenon: ion-triggered change in redox states. This combination of self-assembly, host–guest chemistry, and redox activity provides the potential for the creation of a range of new molecular-scale devices. Since this host is kinetically robust, guest binding in a variety of solvents can be envisaged; its properties in water will be of particular interest; the magnitude of host–guest interactions are usually highly solvent sensitive. Therefore, contrary to conventional Class III systems, it may be possible to tune electronic delocalisation through solvent mixing. The host–guest chemistry of related structures are also currently underway and these studies will form the basis of future reports; in particular the possibility of reversible switching through decomplexation is being investigated.

## Supplementary Material

Supplementary information

Crystal structure data
