# Correction: Modulating the electron-transfer properties of a mixed-valence system through host–guest chemistry

**DOI:** 10.1039/c5sc90026c

**Published:** 2015-05-29

**Authors:** Ahmed Zubi, Ashley Wragg, Simon Turega, Harry Adams, Paulo J. Costa, Vítor Félix, Jim A. Thomas

**Affiliations:** a Department of Chemistry , University of Sheffield , Sheffield , UK . Email: james.thomas@sheffield.ac.uk ; Fax: +44 (0)114 222 9346 ; Tel: +44 (0)114 222 9325; b Biomedical Research Centre , Sheffield Hallam University , Sheffield , S1 1WB , UK; c Departamento de Química , QOPNA and Secção Autónoma de Ciências da Saúde , Universidade de Aveiro , 3810-193 , Aveiro , Portugal; d Departamento de Química , CICECO and Secção Autónoma de Ciências da Saúde , Universidade de Aveiro , 3810-193 , Aveiro , Portugal . Email: vitor.felix@ua.pt ; Fax: +351 234 370 084 ; Tel: +351 234 370 729

## Abstract

Correction for ‘Modulating the electron-transfer properties of a mixed-valence system through host–guest chemistry’ by Ahmed Zubi *et al.*, *Chem. Sci.*, 2015, **6**, 1334–1340.



## 

In the “Electrochemical studies” section of the manuscript, some instances of the terms ‘anodically’ and ‘cathodically’ were erroneously switched. Corrected text of the relevant paragraphs is given below:

“The general changes induced by anion addition on the electrochemistry of **1**^3+^ are most clearly observed using square wave voltammetry and fluoride as a non-redox-active guest – Fig. 6A. As expected, on addition of fluoride, all three Ru^III/II^-based oxidation potentials are shifted cathodically. Interestingly, the response of individual couples is not identical. Up to one equivalent of fluoride causes the first two oxidations to shift into each other, however they separate on further additions, resulting in a maximum Δ*E*_p_ of 100–120 mV, when three equivalents of anion are added, Fig. 6A, Table 3. Further addition of anion produced no additional shifts in oxidation potential until precipitation of the host occurs. Despite analyses for other halide being complicated by the guests' intrinsic redox activity, very different effects were still delineated.

With chloride anions, shifts in host oxidation potentials are virtually over at a 1 : 1 host : guest binding ratio – Fig. 6B, additions of up to a further three equivalents of chloride ion only induce very small additional shifts. This is consistent with the high chloride binding affinity of the macrocycle. A “shoulder” between the second and third oxidation of the host also grows in as chloride is added, this is assigned to the oxidation of chloride, which occurs at 1.08 V in these conditions.^45^ It appears that this couple is broader and slightly anodically shifted compared its free value – this perturbation is likely due to the oxidation of chloride bound to the anionic host as this would be expected to shift in this way. More notably, although the first two Ru^II^ oxidations of the host are cathodically shifted by similar amounts – around 120 mV – the shift for the third oxidation is less half this magnitude.

Any analysis of host-based potential shifts induced by bromide is greatly complicated by the fact that this anion is oxidized in two one-electron steps at 0.765 V and 1.065 V respectively.^46^ So whilst the first host oxidation is clearly defined – being cathodically shifted by 65 mV compared to the free host – the second oxidation is difficult to deconvolute from a bromide-based couple Fig. 6c. However, most strikingly, the third oxidation is clearly *anodically* shifted, suggesting a complex, guest-induced, redistribution of the host's electronic structure.^47–49^”

In addition, the electrochemical shift values reported in Table 3 were reported with the incorrect sign and the table headings were incorrectly displayed; a corrected version of Table 3 appears below:

**Table 3:** Maximum Guest induced electrochemical shifts for **1**^3+^

Halide	Δ*E*_1/2_(**1**)/mV	Δ*E*_1/2_(**2**)/mV	Δ*E*_1/2_(**3**)/mV
F^–^	–100	–120	–110
Cl^–^	–120	–115	–45
Br^–^	–65	∼–100^*a*^	10
I^–^	∼0^*a*^	5	0

^*a*^It is not possible to accurately estimate this value due to a close or overlapping guest-based oxidation couple.

The Royal Society of Chemistry apologises for these errors and any consequent inconvenience to authors and readers.

