# Supplementary material for: Modulating the electron-transfer properties of a mixed-valence system through host–guest chemistry
Source: Chem Sci. 2014 Nov 26;6(2):1334–40. doi: 10.1039/c4sc02799j (PMC5811089; doi:10.1039/c4sc02799j)
Supplement: Supplementary file 1 [file SC-006-C4SC02799J-s001.pdf]

Electronic Supplementary Material (ESI) for Chemical Science.
This journal is © The Royal Society of Chemistry 2014

Supplementary Information for

Modulating the electron-transfer properties of a mixed-valence system through host-guest chemistry.

Ahmed Zubi,[1] Ashley Wragg,[1] Simon Turega,[1] Harry Adams,[1] Paulo J. Costa,[2] Vítor Félix,[3] & Jim A. Thomas[1]

[1]Department of Chemistry, University of Sheffield, Sheffield, UK

[2]Departamento de Química, QOPNA and Secção Autónoma de Ciências da Saúde, Universidade de Aveiro, 3810-193, Aveiro, Portugal

[3]Departamento de Química, CICECO and Secção Autónoma de Ciências da Saúde, Universidade de Aveiro, 3810-193, Aveiro, Portugal

Experimental Details

Crystallographic Determination. Crystals of [**1**](Br_3) were grown by vapor diffusion of diethyl ether into nitromethane solutions. Relevant crystallographic data are summarized in Table S1. X-ray diffraction data were collected at 150 K on a Bruker Smart CCD area detector with Oxford Cryosystem low temperature device. The structure was solved by a combination of direct methods with subsequent difference Fourier syntheses and refined by full matrix least squares on F^2 using SHELX-2013 package.[1] The disordered [9]aneS_3 ligands, one for each independent molecule of $\mathbf{1}^{3+}$, were refined with the methylene groups occupying two alternative positions and refined occupancies of 1-*x*, and *x*, being x equal to 0.726(13) and 0.701(10) for molecules A and B respectively. Hydrogen atoms were placed geometrically and refined with a riding model and with U_{iso} constrained to be 1.2 times U_{eq} of the carrier atom. Molecular diagrams were drawn with Olex2[2] and PyMOL[3] software suites.

CCDC 1004247 contains the supplementary crystallographic data for this paper. These data can be obtained free of charge on application to CCDC, 12 Union Road, Cambridge CB21EZ, UK (fax: (+44) 1223-336-033; e-mail: deposit@ccdc.cam.ac.uk).

DFT calculations. All DFT calculations were performed with the Gaussian09 package[4] with the CAM-B3LYP functional.[5] which as been shown to perform better than B3LYP for a series of properties.[6]

Given the size of the system, a standard 6-31G(d) basis set was used for all elements except for ruthenium and the halogens. The ruthenium was described using the recently developed LANL2TZ(f) basis set[7] with the associated ECP. This is a triple ξ basis set and includes an f polarization function. For fluoride and chloride, the all electron aug-cc-pVDZ was used while for bromide and iodide, the aug-cc-pVDZ-PP with the associated ECP was employed.

In order to try to mimic the conditions of the experimental host:guest studies, the geometry optimizations were performed without constraints in acetonitrile using a polarizable continuum model[8] described with the integral equation formalism variant (IEFPCM) as implemented in Gaussian09.

The molecular electrostatic potential (MEP) and the electron density were calculated from a previously CAM-B3LYP optimized structure of $\mathbf{1}^{3+}$ without the presence of the halogen anions. The representation of the MEP mapped on the density surface was rendered with *PyMOL.*[3]

Table S1 - Crystal data and structure refinement details for [**1**](Br)$_3$

Empirical formula	$C_{36}H_{54}Br_3N_{15}Ru_3S_9$
Formula weight	1528.42
Temperature/K	150(2)
Crystal system	Monoclinic
Space group	$P2_1/c$
a/Å	27.5205(14)
b/Å	21.6096(12)
c/Å	21.7526(12)
β/°	119.123(2)
V /Å^3	11301.0(11)
Z	8
ρ_{calc} mg/mm^3	1.797
μ/mm^{-1}	3.288
F(000)	6048.0
Crystal size/mm^3	0.43 × 0.32 × 0.32
Radiation	MoKα (λ = 0.71073)
2 θ range for data collection	1.7 to 53.96°
Index ranges	$-34 \leq h \leq 30$, $-26 \leq k \leq 27$, $-27 \leq l \leq 27$
Reflections collected	139458
Independent reflections	24312 [R_{int} = 0.0548, R_{sigma} = 0.0631]
Data/restraints/parameters	24312/32/1187
Goodness-of-fit on F^2	1.090
Final R indexes [I ≥2σ (I)]	R_1 = 0.0547, wR_2 = 0.1456
Final R indexes [all data]	R_1 = 0.1128, wR_2 = 0.1645
Largest diff. peak/hole / e Å^{-3}	1.20/-1.85

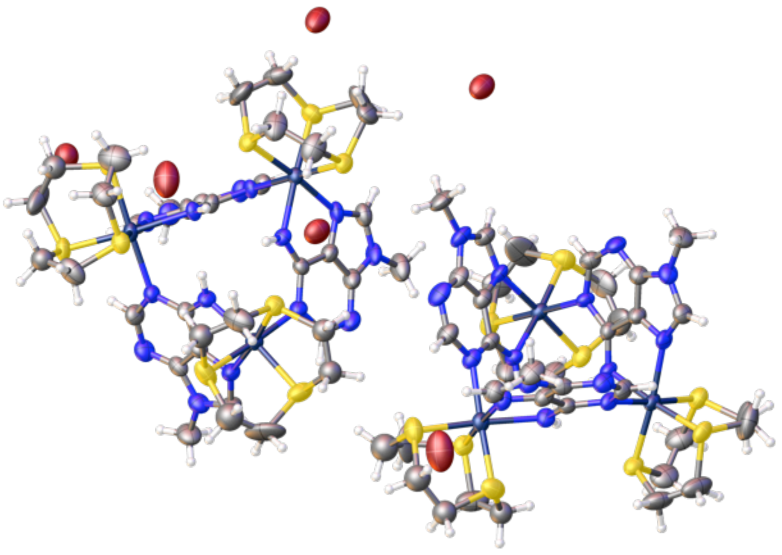

Figure S1. Structure built up from an asymmetric unit composed of two $\mathbf{1}^{3+}$ cations (**A** and **B**) and six bromide counter-ions.

Table S2 **-** X-ray selected bond lengths (Å) and angles (º) around the three octahedral ruthenium centres of [**1**](Br)$_3$ listed together with those obtained for the DFT/CAM-B3LYP optimized structure.*

**Metal centre**	**1**			**2**			**3**		
	**A**	**B**	**DFT**	**A**	**B**	**DFT**	**A**	**B**	**DFT**
Ru-N_7	2.167(5)	2.157(5)	2.154	2.153(4)	2.142(5)	2.153	2.146(5)	2.153(5)	2.155
Ru-N_6H	2.146(5)	2.154(4)	2.156	2.157(4)	2.160(4)	2.156	2.153(4)	2.147(5)	2.155
Ru-N_1	2.147(5)	2.155(5)	2.156	2.134(5)	2.151(4)	2.155	2.141(5)	2.166(5)	2.157
Ru-S_1	2.2812(18)	2.2799(18)	2.364	2.2909(16)	2.2798(17)	2.366	2.2797(18)	2.2799(18)	2.363
Ru-S_4	2.2793(18)	2.2825(15)	2.379	2.2768(17)	2.2768(17)	2.379	2.2809(15)	2.2783(17)	2.381
Ru-S_7	2.2625(15)	2.2809(15)	2.368	2.2886(16)	2.2875(16)	2.367	2.2794(16)	2.2639(15)	2.364
S_1-Ru-N_1	177.48(14)	176.46(13)	177.7	179.22(13)	179.03(13)	177.5	176.42(13)	177.14(13)	177.7
S_4-Ru-N_6H	173.81(13)	175.40(13)	173.5	175.86(13)	175.55(13)	173.3	175.59(13)	174.03(13)	173.8
S_7-Ru-N_7	175.05(14)	174.12(13)	177.1	175.57(13)	175.17(13)	177.3	174.22(13)	175.11(14)	176.7
N_6H-Ru-N_7	79.72(18)	79.80(16)	79.5	79.95(17)	79.21(18)	79.5	79.98(17)	79.80(17)	79.6

*The atomic notation scheme followed is depicted in Scheme 1.

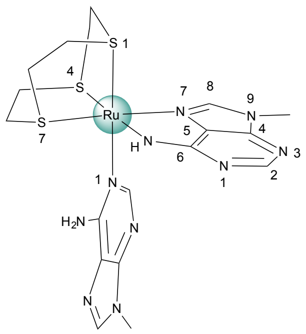

Scheme S1. Atomic notation scheme adopted for [9]aneS_3 and 9-methyladenine ligands in Table 1, using IUPAC nomenclature.

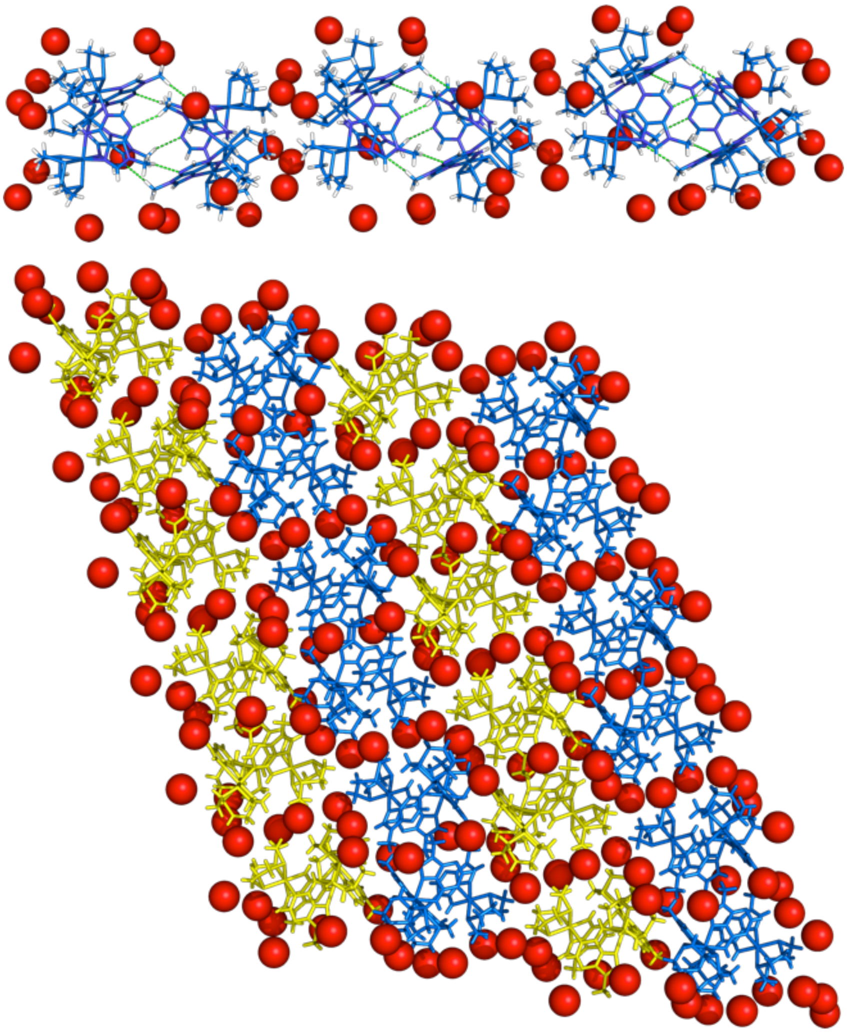

Figure S2. Crystal packing view along the *b* crystallographic direction showing strands of alternating **A** and **B** bowls surrounded by bromide anions, that make a number of close C-H···Br^- contacts with methylenic residues of coordinated thiacrowns. Top, a single strand composed by dimers of molecules **A** (in blue) and surrounded by bromide anions (in red). Bottom, strands of molecules **A** packed in alternate fashion with strands of molecules **B** (in yellow).

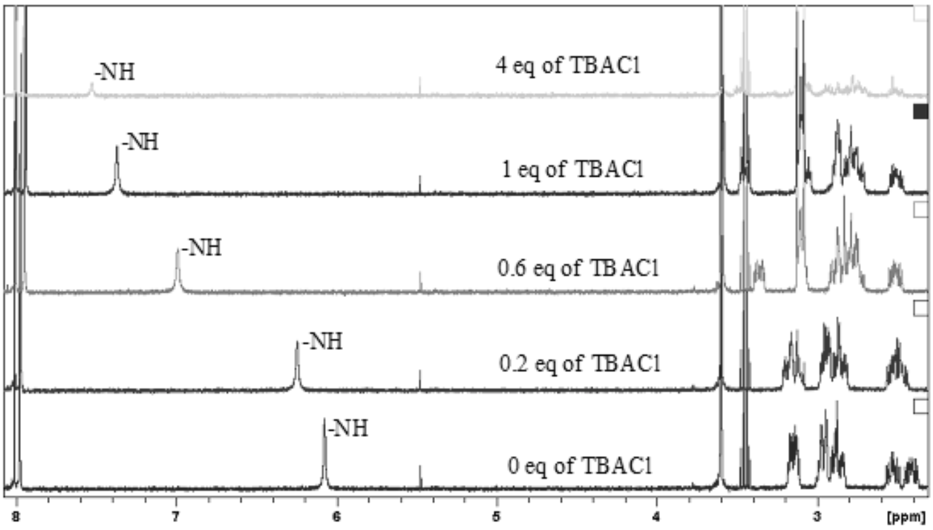

Figure S3. Detail of ^{1}H-NMR spectral changes in the NH6 protons of the 9MA bridging ligand of $\mathbf{1}^{3+}$ upon addition of TBACl. Conditions: 400 MHz, Solvent CD_3CN; temperature: 293 K; [$\mathbf{1}^{3+}$] =1.5×10^{-6}mol dm^{-3}.

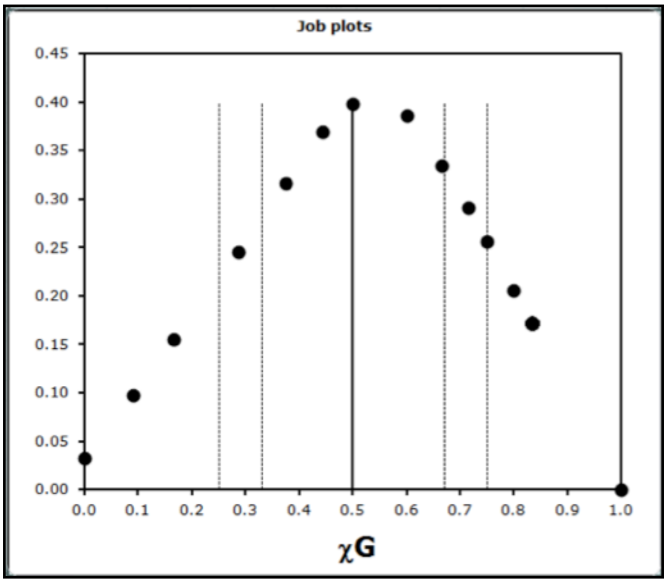

Figure S4. Job-plot analysis based on ^{1}H-NMR spectral changes in the NH6 protons on addition of chloride

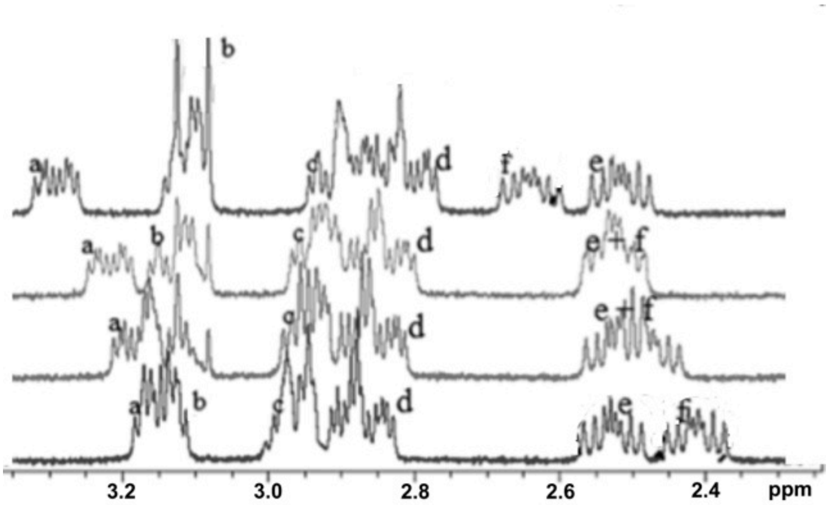

Figure S5. ^{1}H-NMR spectral changes in thiacrown ligand signals of $\mathbf{1}^{3+}$ upon addition of TBACl. Conditions: 400 MHz, Solvent CD_3CN; temperature: 293 K; [**1**] =1.5×10^{-6}mol dm^{-3}.

.

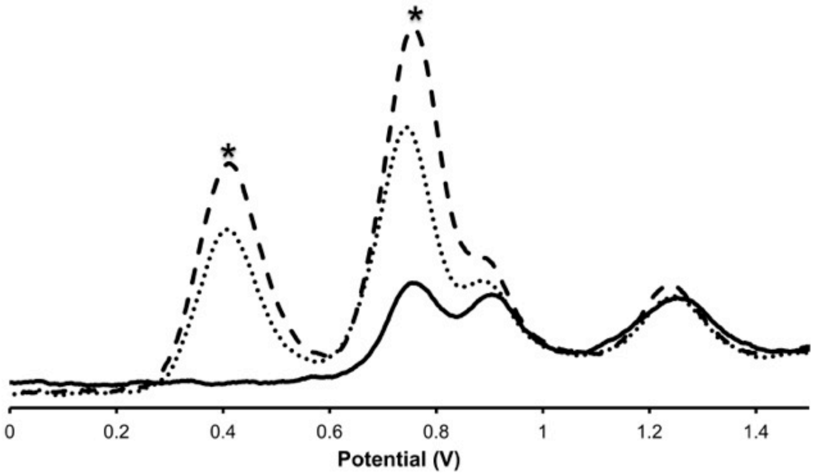

Figure S6. Square wave voltammograms, SWV, for the oxidations of $\mathbf{1}^{3+}$ on the addition of (A) TBAI. The peaks marked * are due to oxidations associated with the iodide guest.

References

[1] G. Sheldrick. A short history of SHELX. Acta Cryst. 2008. A64:112-122.

[2] O. V. Dolomanov, L. J. Bourhis, R. J. Gildea, J. A. K. Howard and H. Puschmann, *OLEX2*: a complete structure solution, refinement and analysis program, *J. Appl. Cryst.* (2009). 42, 339-341.

[3] W. L. Delano, *The PyMOL Molecular Graphics System* 2002, *Version 1.2r2, Schrödinger, LLC.*

[4] – Frisch, M. J.; Trucks, G. W.; Schlegel, H. B.; Scuseria, G. E.; Robb, M. A.; Cheeseman, J. R.; Zakrzewski, V. G.; Montgomery, Jr., J. A.; Stratmann, R. E.; Burant, J. C.; Dapprich, S.; Millam, J. M.; Daniels, A. D.; Kudin, K. N.; Strain, M. C.; Farkas, O.; Tomasi, J.; Barone, V.; Cossi, M.; Cammi, R.; Mennucci, B.; Pomelli, C.; Adamo, C.; Clifford, S.; Ochterski, J.; Petersson, G. A.; Ayala, P. Y.; Cui, Q.; Morokuma, K.; Rega, N.; Salvador, P.; Dannenberg, J. J.; Malick, D. K.; Rabuck, A. D.; Raghavachari, K.; Foresman, J. B.; Cioslowski, J.; Ortiz, J. V.; Baboul, A. G.; Stefanov, B. B.; Liu, G.; Liashenko, A.; Piskorz, P.; Komaromi, I.; Gomperts, R.; Martin, R. L.; Fox, D. J.; Keith, T.; Al-Laham, M. A.; Peng, C. Y.; Nanayakkara, A.; Challacombe, M.; Gill, P. M. W.; Johnson, B.; Chen, W.; Wong, M. W.; Andres, J. L.; Gonzalez, C.; Head-Gordon, M.; Replogle, E. S.; Pople, J. A. Gaussian 09, Revision A.01; Gaussian, Inc.: Wallingford, CT, 2009.

[5] T. Yanai, D. Tew, and N. Handy, "A new hybrid exchange-correlation functional using the Coulomb-attenuating method (CAM-B3LYP)," Chem. Phys. Lett., 393 (2004) 51-57.

[6] M. J. G. Peach, T. Helgaker, P. Sałek, T. W. Keal, O. B. Lutnæs, D. J. Tozer, N. C. Handy Phys. Chem. Chem. Phys., 2006, 8, 558–562.

[7] Roy, L. E.; Hay, P. J.; Martin, R. L. J. Chem. Theory Comput. 2008, 4, 1029.

[8] Barone, V.; Cossi, M.; Tomasi, J. J. Chem. Phys. 2002, 107, 43−54.
